# Contact tracing using a real-time location system in a tertiary-care hospital in Singapore

**DOI:** 10.1017/ash.2023.372

**Published:** 2023-09-29

**Authors:** Guan Yee Ng, Biauw Chi Ong

## Abstract

**Background:** Densely populated metropolitan cities like Singapore are susceptible to emerging infectious disease (EID) outbreaks. Singapore’s pandemic control measures include running biennial simulation exercises for all public hospitals on EID case management, in which a key assessment criterion is contact tracing. Current methods of contact tracing that involve retrospective review of the electronic medical record (EMR) are time-consuming and heavily manpower dependent, and they fail to capture a significant number of contacts. A real-time location system (RLTS) was accurate and effective in contact tracing. We compared the time taken to perform contact tracing and list of contacts identified for RTLS versus EMR, and we compared manpower and manpower hours required to perform contact tracing for RTLS versus EMR. Then we extrapolated the cost incurred by RTLS versus EMR. **Methods:** A prospective case study was conducted during a simulation exercise to determine and compare the list of contacts, time required, manpower required, and manpower hours required between RTLS and EMR. The costs of both methods were also compared. **Results:** RTLS identified almost 3 times more contacts than EMR (Fig. 2) with a 96.2% reduction in time taken, a 97.6% reduction in manpower, and a 97.5% reduction in manpower hours (Fig. 1). RTLS incurred significant equipment cost and therefore might require many contact-tracing episodes before providing economic benefit (Fig. 3). However, its speed and accuracy provided during contact tracing will allow the hospital to quickly isolate potentially exposed contacts, reducing the number of infected people during the spread of an infectious disease, particularly one like COVID-19. **Conclusions:** Albeit costly, RTLS is effective at contact tracing. RTLS has the potential to be the gold standard in contact-tracing methods of the future, particularly considering the current pandemic.

**Figure:**
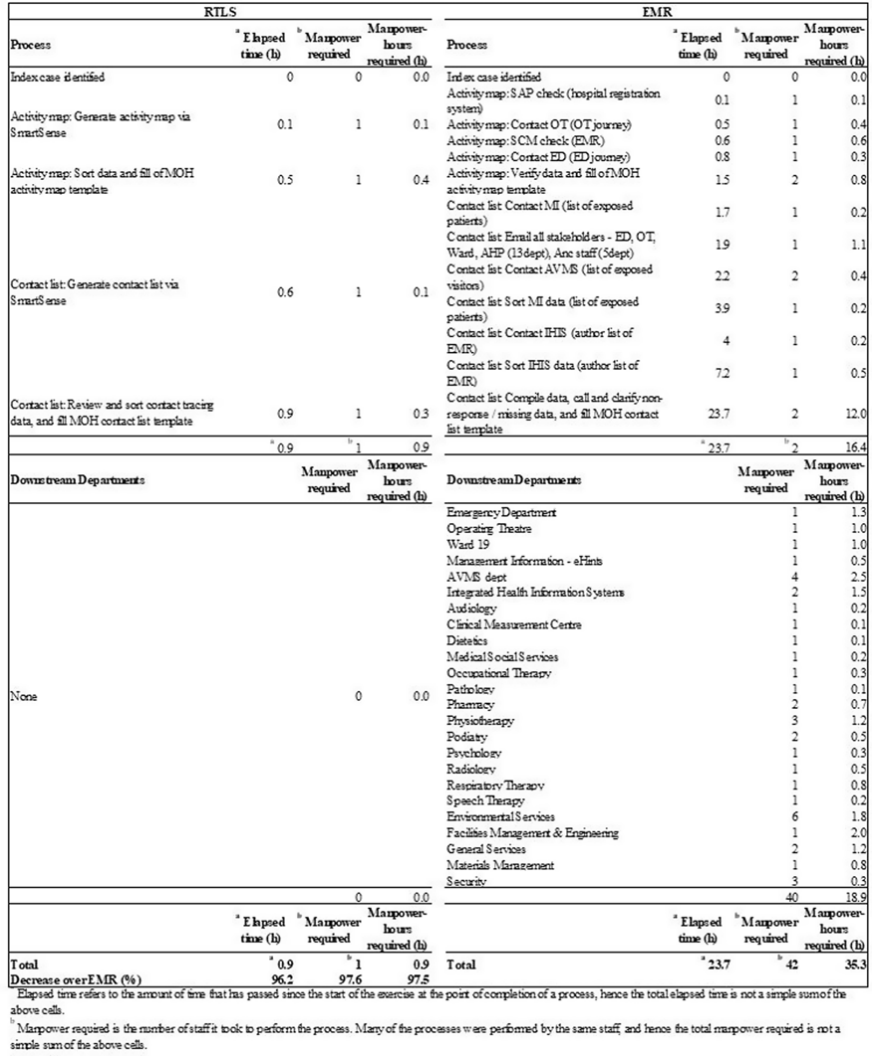

**Figure:**
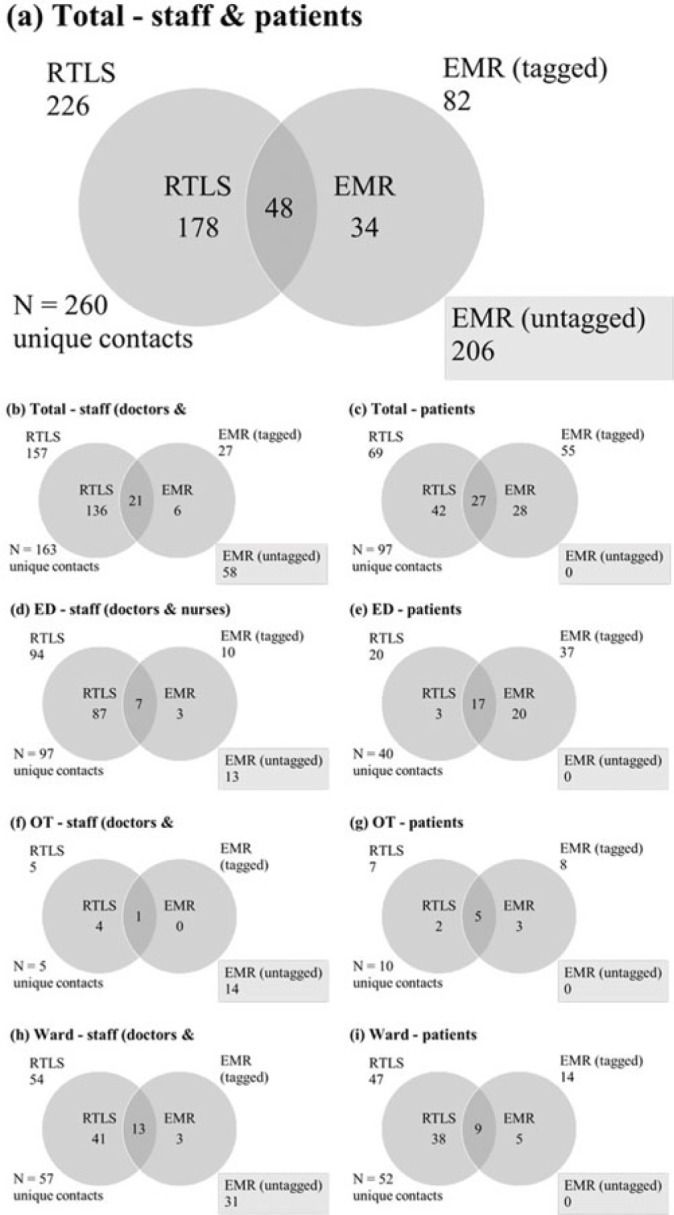

**Figure:**
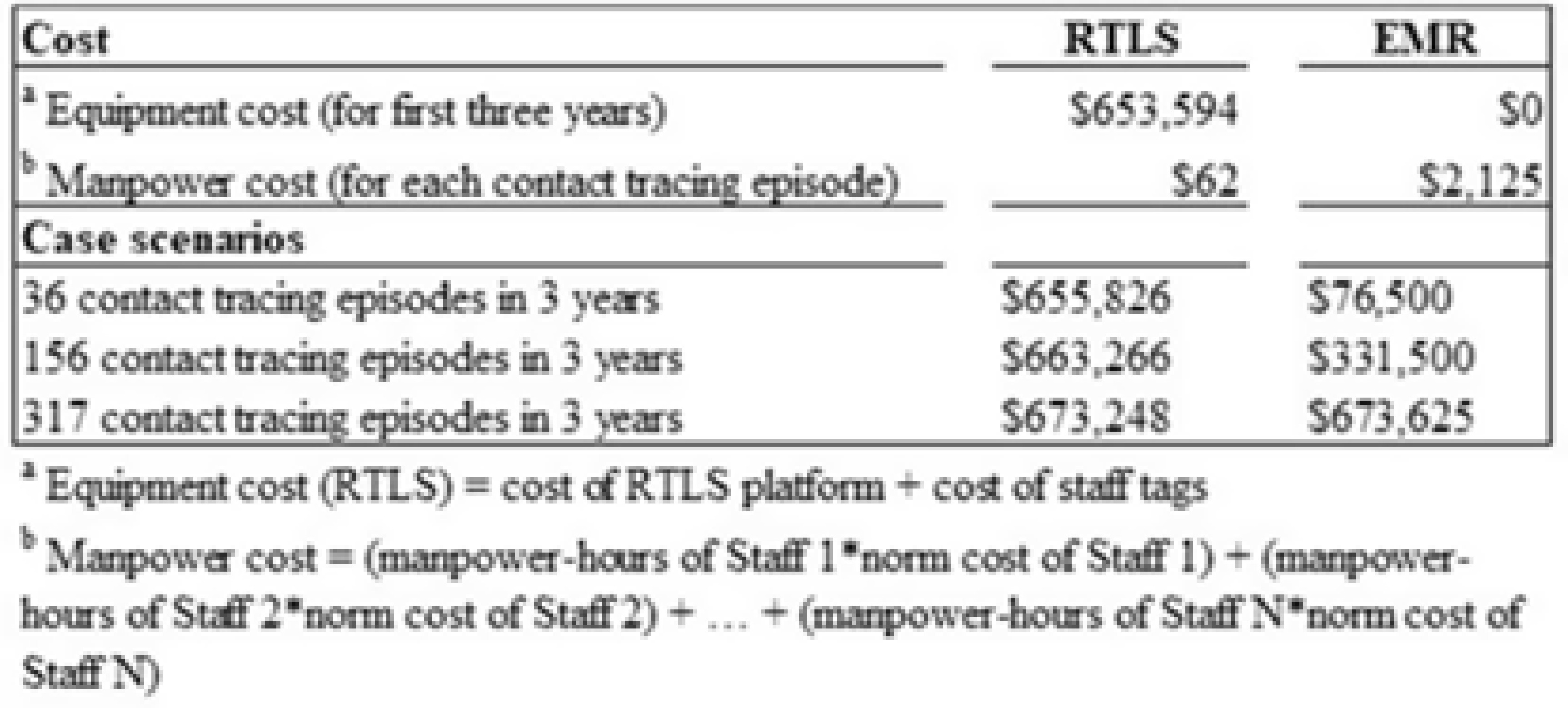

**Disclosures:** None

